# DNA methylation episignature and comparative epigenomic profiling for Pitt-Hopkins syndrome caused by *TCF4* variants

**DOI:** 10.1016/j.xhgg.2024.100289

**Published:** 2024-04-02

**Authors:** Liselot van der Laan, Peter Lauffer, Kathleen Rooney, Ananília Silva, Sadegheh Haghshenas, Raissa Relator, Michael A. Levy, Slavica Trajkova, Sylvia A. Huisman, Emilia K. Bijlsma, Tjitske Kleefstra, Bregje W. van Bon, Özlem Baysal, Christiane Zweier, María Palomares-Bralo, Jan Fischer, Katalin Szakszon, Laurence Faivre, Amélie Piton, Simone Mesman, Ron Hochstenbach, Mariet W. Elting, Johanna M. van Hagen, Astrid S. Plomp, Marcel M.A.M. Mannens, Mariëlle Alders, Mieke M. van Haelst, Giovanni B. Ferrero, Alfredo Brusco, Peter Henneman, David A. Sweetser, Bekim Sadikovic, Antonio Vitobello, Leonie A. Menke

**Affiliations:** 1 Department of Human Genetics, Amsterdam University Medical Centers, University of Amsterdam, Amsterdam, the Netherlands; 2 Amsterdam Reproduction & Development, Amsterdam, the Netherlands; 3 Verspeeten Clinical Genome Centre, London Health Science Centre, London, ON, Canada; 4 Department of Pathology and Laboratory Medicine, Western University, London, ON, Canada; 5 Department of Medical Sciences, University of Torino, Torino, Italy; 6 Amsterdam UMC location University of Amsterdam, Emma Children’s Hospital, Department of Pediatrics, Amsterdam, the Netherlands; 7 Zodiak, Prinsenstichting, Purmerend, the Netherlands; 8 Department of Clinical Genetics, Leiden University Medical Center, Leiden, the Netherlands; 9 Department of Human Genetics, Donders Institute for Brain, Cognition and Behaviour, Radboud University Medical Center, Nijmegen, the Netherlands; 10 Department of Human Genetics, Radboud University Medical Center, Nijmegen, the Netherlands; 11 Department of Human Genetics, Friedrich-Alexander-Universität Erlangen-Nürnberg (FAU), Erlangen, Germany; 12 Department of Human Genetics, University of Bern, Inselspital Universitätsspital Bern, Bern, Switzerland; 13 Institute of Medical and Molecular Genetics (INGEMM), La Paz University Hospital, Madrid, Spain; 14 Institute for Clinical Genetics, University Hospital Carl Gustav Carus at the Technische Universität Dresden, Dresden, Germany; 15 Institute of Paediatrics, Faculty of Medicine, University of Debrecen, Debrecen, Hungary; 16 UFR Des Sciences de Santé, INSERM-Université de Bourgogne UMR1231 GAD «Génétique des Anomalies du Développement», FHUTRANSLAD, Dijon, France; 17 CHU Dijon Bourgogne, Centre de Génétique, Centre de Référence Maladies Rares «Anomalies du Développement et Syndromes Malformatifs», FHU-TRANSLDAD, Dijon, France; 18 Genetic Diagnosis Laboratories, Strasbourg University Hospital, Strasbourg 67000, France; 19 Swammerdam Institute for Life Sciences, FNWI, University of Amsterdam, Amsterdam, the Netherlands; 20 Department of Public Health and Pediatrics, University of Torino, Turin, Italy; 21 Department of Medical Sciences, University of Torino, Turin, Italy; 22 Division of Medical Genetics and Metabolism and Center for Genomic Medicine, Massachusetts General for Children, Boston, MA, USA; 23 Unité Fonctionnelle Innovation en Diagnostic Génomique des Maladies Rares, FHU-TRANSLAD, CHU Dijon Bourgogne, Dijon, France; 24 Amsterdam Neuroscience - Cellular & Molecular Mechanisms, Amsterdam, the Netherlands

**Keywords:** Pitt-Hopkins syndrome, PTHS, *TCF4*, neurodevelopmental disorder, DNA methylation, episignature, CNV, VUS

## Abstract

Pitt-Hopkins syndrome (PTHS) is a neurodevelopmental disorder caused by pathogenic variants in *TCF4*, leading to intellectual disability, specific morphological features, and autonomic nervous system dysfunction. Epigenetic dysregulation has been implicated in PTHS, prompting the investigation of a DNA methylation (DNAm) "episignature" specific to PTHS for diagnostic purposes and variant reclassification and functional insights into the molecular pathophysiology of this disorder. A cohort of 67 individuals with genetically confirmed PTHS and three individuals with intellectual disability and a variant of uncertain significance (VUS) in *TCF4* were studied. The DNAm episignature was developed with an Infinium Methylation EPIC BeadChip array analysis using peripheral blood cells. Support vector machine (SVM) modeling and clustering methods were employed to generate a DNAm classifier for PTHS. Validation was extended to an additional cohort of 11 individuals with PTHS. The episignature was assessed in relation to other neurodevelopmental disorders and its specificity was examined. A specific DNAm episignature for PTHS was established. The classifier exhibited high sensitivity for *TCF4* haploinsufficiency and missense variants in the basic-helix-loop-helix domain. Notably, seven individuals with *TCF4* variants exhibited negative episignatures, suggesting complexities related to mosaicism, genetic factors, and environmental influences. The episignature displayed degrees of overlap with other related disorders and biological pathways. This study defines a DNAm episignature for TCF4-related PTHS, enabling improved diagnostic accuracy and VUS reclassification. The finding that some cases scored negatively underscores the potential for multiple or nested episignatures and emphasizes the need for continued investigation to enhance specificity and coverage across PTHS-related variants.

## Teaser

Unveiling a DNA methylation signature specific to Pitt-Hopkins syndrome (PTHS), this study enhances diagnostic accuracy and variant reclassification. The signature shows high sensitivity for TCF4 variants, with complexities like mosaicism emphasized. This finding underscores the need for continued research to enhance specificity and coverage across PTHS-related variants.

## Main text

Pitt-Hopkins syndrome (PTHS; OMIM: 602272) is a rare neurodevelopmental disorder associated with developmental delays with moderate to severe intellectual disability, distinctive facial features, gastrointestinal problems, and breathing regulation anomalies that are at least in part related to autonomic nervous system dysfunction.^1^^,^^2^ Additional common neurodevelopmental features include autism spectrum disorder and seizures. The clinical diagnosis of PTHS relies on recently published clinical diagnostic criteria.^1^ Molecular confirmation of PTHS involves the identification of heterozygous single-nucleotide or insertion-deletion variants with a loss-of-function effect or structural variants disrupting *TCF4*, encoding transcription factor 4, located in e18q21.2.^3^^,^^4^

TCF4 is a basic-helix-loop-helix (bHLH) transcription factor that regulates gene expression through homodimerization or by heterodimerization with other transcription factors belonging or not to the bHLH family and binding to specific DNA regulatory sequences (CANNTG) known as Ephrussi boxes.^5^^,^^6^ Generally, these transcription factors do not function individually or in pairs but are usually part of a large transcriptional machinery comprising all sorts of, but not limited to, transcription factors, RNA polymerases, adaptor proteins, coactivators, and epigenetic regulators.^7^ Epigenetic regulators add an extra layer of gene expression regulation by altering the chromatin state of DNA by adding or removing specific epigenetic marks (e.g., methylation, acetylation) directly on the DNA or via histone modifications.^8^ It is possible that the transcriptional complex surrounding TCF4 contains epigenetic regulators, as it was shown that heterozygous loss of function of *Tcf4* alters the CpG methylation state in cells of the murine hippocampus, suggesting that this transcriptional complex can alter DNA methylation (DNAm). This may take place directly through its interaction partners or indirectly by regulating the expression of proteins involved in the regulation of DNAm (e.g., DNA methyltransferases).^8^

Previous research demonstrated that individuals with anomalies in the epigenetic machinery display syndrome-specific array-based DNAm patterns known as “episignatures.”^9^ Episignatures have emerged as sensitive biomarkers in diagnostics for various neurodevelopmental disorders^10^ and are particularly useful for reclassifying genetic variants of uncertain significance (VUSs).^11^ Given the link between *TCF4* and DNAm,^12^ our study aimed to derive a PTHS-specific DNAm episignature for diagnostic purposes.

For this purpose, we collected DNA samples from peripheral blood cells of individuals with molecularly confirmed PTHS or with VUSs in *TCF4*. Clinical characteristics, including the clinical diagnostic criteria^1^ of the participants, are detailed in the Table S1. Informed consent was obtained from all participants or their caretakers, and the study adhered to the principles of the Declaration Helsinki. Approval was obtained from local institutional review boards (Amsterdam UMC, UAB22-053; Western University, REB116108 and REB106302; Dijon University Hospital, DC2011-1332).

A total of 78 individuals with PTHS carrying pathogenic, likely pathogenic, or variants of unknown significance in *TCF4* were included. Among them, 23 individuals carried a missense variant, 17 a copy-number variant (CNV) partially or completely encompassing *TCF4*, fifteen a frameshift variant, 10 a nonsense variant, 11 a splice site variant, one a synonymous variant (results in a loss-of-function effect), and one a chromosomal translocation with its breakpoint in *TCF4*. Variants were classified by the diagnostic labs according to the classification guidelines of the American College of Medical Genetics (ACMG) and Association for Molecular Pathology (AMP)^13^^,^^14^ using GRCh37 NM_001083962.2 for annotation. Molecular details of the cohort are provided in Table S2. All individuals either had a clinical diagnosis of PTHS or presented with symptoms belonging to the clinical spectrum associated with PTHS.

**Figure 1:**
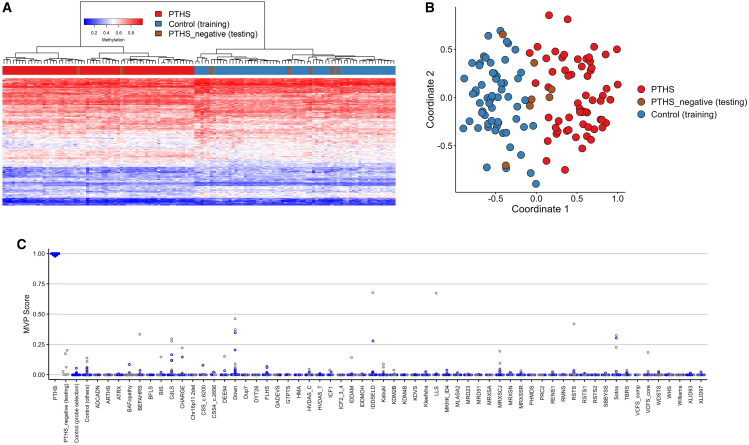
PTHS episignature discovery cohort and negative cases (A) Hierarchical clustering heatmap. Each column in the heatmap represents an individual from the TCF4 discovery case group (*n* = 60), the negative case group (*n* = 7), or the discovery control group. Meanwhile, each row corresponds to a probe that has been specifically selected for the PTHS episignature. The heatmap depicts Euclidean clustering, revealing a distinct separation between the TCF4 discovery cases in red and the control cases in blue. Negative cases, shown in brown, cluster with controls, with two exceptions. (B) Multidimensional scaling (MDS) plot. This plot visually presents the segregation of individuals with TCF4 and controls through MDS analysis. (C) Support vector machine (SVM) classifier model scores. The SVM classifier model scores are depicted. The model was trained using the selected PTHS episignature probes, with 75% of controls and 75% of individuals with other neurodevelopmental disorders (depicted in blue). The remaining 25% of controls and 25% of samples from other disorders were used for testing and are displayed in gray. The plot demonstrates that all individuals with PTHS exhibited MVP scores >0.75. Conversely, all negative individuals displayed an MVP score <0.25, indicative of the absence of the PTHS episignature.

To generate the PTHS episignature,^10^^,^^11^ the 78 participants were randomly divided into a discovery cohort (67/78 individuals) and a validation cohort (11/78 individuals) and supplied to the EpiSign Discovery Pipeline.^10^ Bisulfite-treated DNA from peripheral blood cells of discovery cohort individuals was analyzed with the Infinium Methylation EPIC BeadChip array (San Diego, CA, USA) and compared to 85 age- and sex-matched controls (for comprehensive methodology, see the supplemental methods). Primary analyses included all cases with variants in *TCF4* (cases 1–67). After conducting multiple rounds of classifier probe selection and MVP scoring, we employed unsupervised hierarchical clustering and MDS approaches. Through this analysis, we identified seven cases (61–67) with low MVP scores. Interestingly, these cases clustered with controls rather than with the PTHS group, as illustrated in Figure 1. This suggests that their DNA methylation profiles are distinct from those typically associated with PTHS. To proceed with probe selection and construction of the PTHS episignature classifier, these samples were removed from the discovery cohort, and the remaining cohort of 60 cases with similar DNAm profiles was used as the discovery cohort (supplemental methods). 102 differentially methylated positions (DMPs) were selected (Table S3), allowing for complete separation of individuals with PTHS from controls in the training cohort (Figure S1). Finally, 20 rounds of leave-25%-out cross-validation were performed to test the validity of the classifier. All discovery cohort samples clustered together with cross-validation training cases, demonstrating the robustness and sensitivity of the episignature for this cohort (Figure S2). Using a cutoff of 0.25 for the MVP score, the model’s specificities for the *TCF4* episignature are 100% (relative, 99.41%, the sets of unaffected controls (individuals with no known rare genetic disorder or pathogenic or unknown significance variant), unresolved cases (those suspected to have genetic disorders but with no definitive genetic or EpiSign diagnosis), respectively.

**Figure 2:**
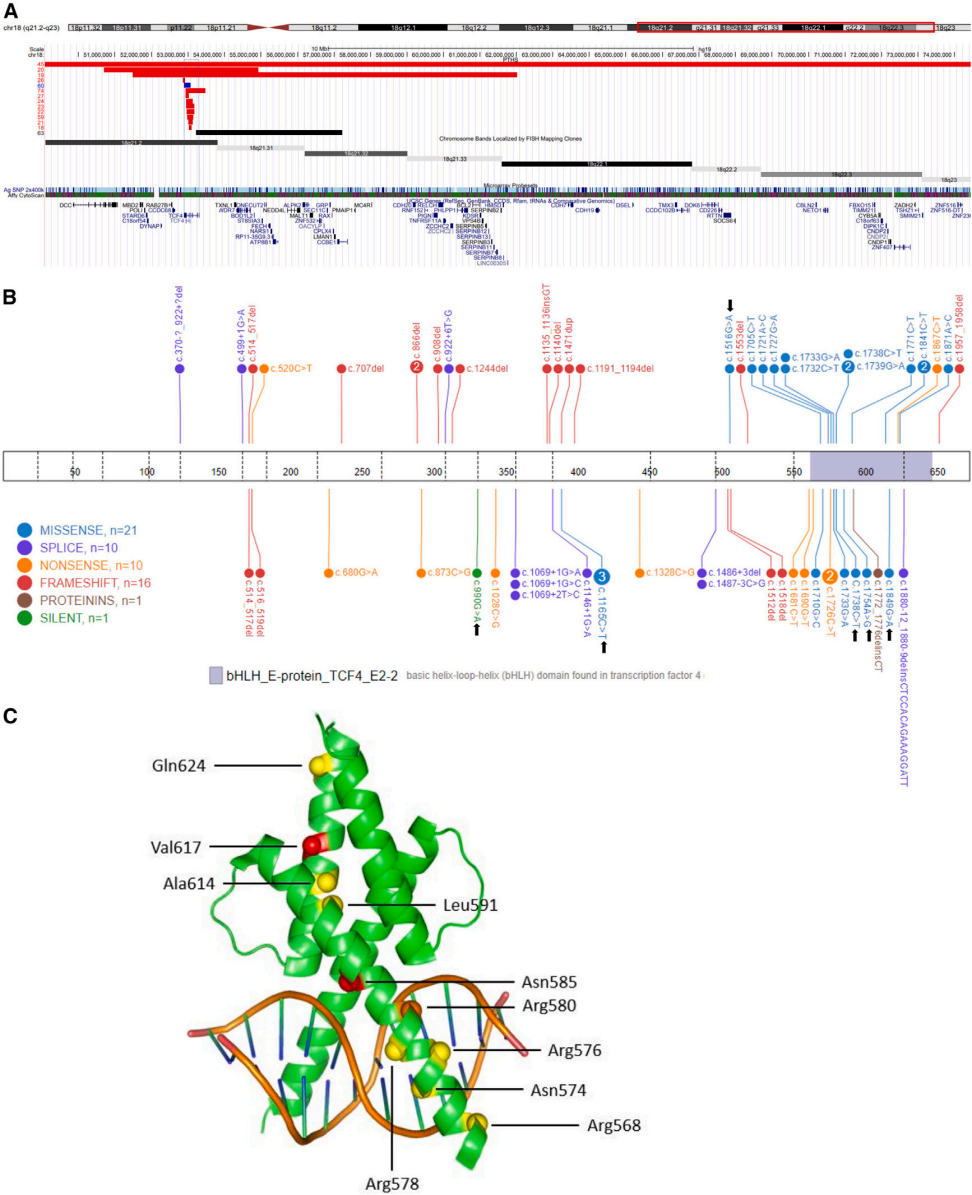
Overview of CNVs and TCF4 variants in the study cohort (A) Chromosomal structural variations. Chromosome 18q alterations are visualized through horizontal bars: red bars depict large deletions, blue bars indicate duplications, and the black bar signifies the CNV linked to an absent PTHS episignature. Genes encompassed within this region are listed below. Cytogenetics banding and recognized genes were sourced from the UCSC Genome Browser 2009 (GRCh37/hg19) genome build. (B) TCF4 variants. A comprehensive summary of TCF4 variants within the cohort is presented. Variants identified by black arrows demonstrated negative PTHS episignature outcomes. Notably, for the recurrent c.1738C>T (p.Arg580Trp) variant, one participant displayed an absent PTHS episignature, while two individuals exhibited a positive PTHS episignature. The visualization was created using the St. Jude Cloud Protein Paint Image tool at https://pecan.stjude.cloud/proteinpaint. (C) TCF4 basic-helix-loop-helix (bHLH) domain. The (homodimerized) TCF4 bHLH domain, in complex with the Ephrussi box (E-box) DNA element, is depicted through PyMOL visualization (PDB: 6OD5, https://doi.org/10.2210/pdb6OD5/pdb). Amino acid residues impacted by missense variants within this domain are denoted with spheres. A color code is used: yellow signifies residues associated with a positive PTHS episignature, red indicates a negative PTHS episignature, and orange indicates equivocal results (variant c.1738C>T [p.Arg580Trp]).

The molecular details of the PTHS cohort in this study are summarized in Table S2 and Figure 2.

**Figure 3:**
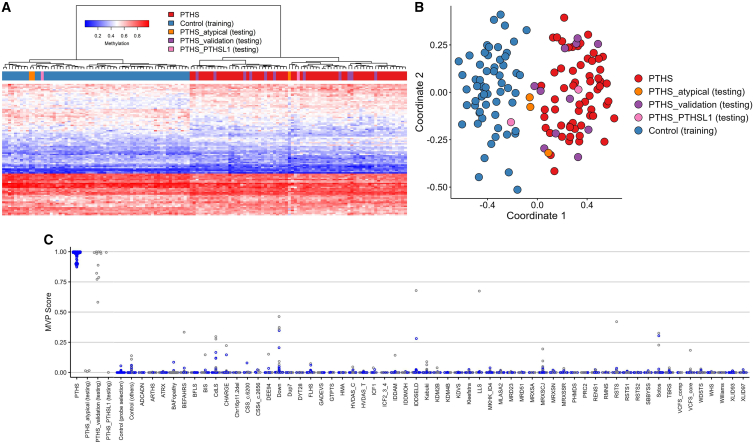
dAssessment of the PTHS episignature (A) Hierarchical clustering heatmap. Each column represents an individual with PTHS or control, while each row corresponds to a probe selected for the episignature. The heatmap visually depicts a distinct separation between individuals with PTHS (highlighted in red and pink) used for training and validation and controls (depicted in blue). Notably, all but one PTHS_atypical individual (in orange) are closely aligned with control cases. Similarly, one of the PTHS_PTHSL1 individuals (in purple) maps with the patient cluster, while the other aligns with controls. (B) MDS plot. The plot demonstrates the pronounced distinction between PTHS discovery and validation individuals (in red and pink, respectively), which were utilized for training, and the control group (in blue). This separation confirms the efficacy of the episignature in distinguishing individuals with PTHS from controls. Similar to the hierarchical clustering, all PTHS_atypical cases but one (in orange) share proximity with control cases. Furthermore, one of the PTHS_PTHSL1 individuals (in purple) displays an association with the patient cluster, while the other corresponds to controls. (C) SVM classifier model. The SVM model was trained employing the selected PTHS episignature probes and a cohort comprising 75% of controls and 75% of other neurodevelopmental disorder samples (in blue). The remaining 25% of controls and neurodevelopmental disorder samples were reserved for testing (in gray). All validation samples clustered with PTHS and had high MVP scores. One PTHS_atypical individual and one PTHS_PTHSL1 individual clustered with training and validation individuals. Conversely, the remaining two PTHS_atypical individuals and one PTHS_PTHSL1 individual displayed an MVP score <0.25 and clustered with controls.

As the next step in episignature generation, the PTHS DNAm classifier was validated with DNAm profiles of 11 additional individuals with PTHS (participants 68–78), of whom four carried a frameshift variant, two had a splice site variant, two had a missense variant, two had a *TCF4* nonsense variant, and one had a CNV covering *TCF4* (Table S2). DNAm profiles were subjected to hierarchical clustering and MDS, confirming that all randomly selected validation samples clustered together with the discovery cohort. Using the DNAm classifier, all validation samples scored an MVP >0.5, indicating the presence of the PTHS episignature (Figure 3).

To enhance the robustness of the PTHS episignature, an additional round of probe selection was conducted wherein validation samples were included in the discovery cohort. All samples clustered together and had high MVP scores (MVP ≈1) (Figure S3), and we obtained a final list of 164 differentially methylated probes. To test the validity, we performed 20 rounds of leave-25%-out cross-validation with all 71 samples, revealing the robustness and sensitivity of the episignature for the full cohort (Figure S4).

The molecular and clinical data of the seven individuals with negative PTHS episignatures (Figure 2; Table S1) were closely inspected to infer the cause of the negative results.

Participant 64 had a possible diagnosis of PTHS; however c.1516G>A (p.Val506Ile) affects a moderately conserved amino acid (PhyloP100:4.601), and *in silico* predictors are associated with moderate to strong benign Meta scores, including REVEL = 0.106 and BayesDel addAF = −0.2059 (Varsome). The inheritance of the variant is unknown for this patient. This allele has been reported one time in gnomAD (v.4.0.0). This variant had been considered as a VUS before episignature investigation.

Participant 65 had insufficient clinical evidence for conclusive clinical diagnosis of PTHS. The *de novo* c.1754A>G (p.Asn585Ser) variant is not cataloged in gnomAD or variant archive ClinVar. This variant affects a highly conserved amino acid (PhyloP100:8.017) and was associated with supporting to strong pathogenic Meta scores including REVEL = 0.955 and BayesDel addAF = 0.2839. It was previously classified as likely pathogenic before subjecting it to episignature investigation.

Two cases that were negative for the PTHS episignature carried a CNV with MVPs scores of 0.17 and 0.20, respectively. Participant 66 had a *de novo* deletion (arr[GRCh37]18q21.2q21.32(53232878–57234972)x1), which covers the 5′ UTR, the transcription start site, and the first two exons of some (long) *TCF4* mRNA transcripts. Other (shorter) *TCF4* transcripts are unaffected. A possible explanation for the absence of the PTHS episignature could therefore be rescue of TCF4 function by products of unaffected *TCF4* transcripts. Indeed, shorter downstream transcripts of TCF4 appear to be upregulated during neuronal differentiation.^15^ Based on clinical diagnostic criteria, this individual had a possible diagnosis of PTHS; thus, the phenotype could hypothetically also be caused by other deleted genes in the CNV region or a currently unidentified genetic aberration. Participant 61 carried a 12 Mb mosaic deletion 18q21.1–18q22.2 (including *TCF4*), estimated to be present in ∼25% of (blood) cells. There was a clinical diagnosis of PTHS, with very specific phenotypic signs (Table S1). This inconsistency may be attributed to the limited sensitivity of episignature analysis in the context of tissue mosaicism.^16^^,^^17^ Therefore, the absence of the PTHS episignature in participant 61 does not rule out pathogenicity of this CNV.

Three cases carrying the pathogenic variants, 62 (c.1738C>T [p.Arg580Trp]), 63 (c.990G>A [p.Ser330=]), and 67 (c.1849G>A [p.Val617Ile]), showed no evidence of the PTHS episignature within the defined parameters in the rest of the cohort (Figure 2). The variants p.Arg580Trp and p.Val617Ile have previously been reported in other individuals with PTHS, and p.Arg580Trp shows a strong association with pronounced PTHS^18^^,^^19^ (also observed in the present cohort in individuals 5 and 10), while p.Val617Ile was associated with a mild disease phenotype in one girl.^20^ Participant 63 (p.Ser330=) had a diagnosis of PTHS (Table S1). Multiple lines of evidence supported the likely pathogenic role of the *de novo* variant c.990G>A (p.Ser330=) cataloged in ClinVar and in the curated database ClinGen. In particular, this variant has been reported as a *de novo* occurrence in multiple affected individuals with intellectual disability^19^^,^^21^ (ACMG/AMP criteria PS2, PM6, PS4_supporting). It is absent from gnomAD (PM2_supporting), and splice prediction analysis using multiple computational tools suggests an impact on splicing (PP3). mRNA sequencing analysis in primary fibroblasts obtained from participant 63 suggested a milder pathogenic effect of this variant resulting in the residual detection of mutated transcripts containing exon skipping (15%) or intronic retention (27%), as well as normal RNA splicing (8%). The fraction of missing transcripts affected by nonsense-mediated RNA decay was estimated to be 50%. Overall, the functional data support the synonymous c.990G>A (p.Ser330=) variant as resulting in a loss-of-function effect on about the 42% of the transcripts (data not shown).

Although the episignature below the cutoff in participant 67 (p.Val617Ile) may potentially be explained by a hypomorphic variant, the finding of a negative PTHS episignature in participant 62, carrying a variant associated with a positive PTHS episignature in other individuals in the present cohort, and in participant 63, carrying a variant leading to a splicing defect, was unexpected and suggests different confounding factors. A possible explanation for this contradictory finding could be attributed to the influence of genetics and environmental factors on DNAm patterns.^9^ For example, in the current analysis, we cannot exclude the possibility of additional variants confounding the PTHS DNAm profile in individuals with a PTHS-negative episignature. Another factor that could explain this observation is the effect of an (unknown) environmental effect on DNAm, such as the one observed in fetal alcohol syndrome.^22^

It can be inferred that negative PTHS episignature outcomes may be attributable to factors such as benign or moderate variant effects, mosaicism, or the interplay of genetic and environmental influences affecting DNAm profiles. Therefore, it is important to bear in mind these potential pitfalls in the interpretation of episignatures, particularly regarding DNAm-related confounding factors and especially in the context of negative results in individuals with clinical PTHS.^11^ To improve diagnostic accuracy, further studies will be needed to model the influence of genetics and environment on episignatures.

To assess the specificity of the PTHS episignature in the context of *TCF4* diagnostics, we investigated DNAm profiles of three cases (participants 79–81) with the *de novo* likely pathogenic *TCF4* variant c.1165C>T (p.Arg389Cys). This variant was associated with moderate to severe intellectual disability, language impairment, and non-specific facial dysmorphisms in six individuals with insufficient clues for PTHS, according to diagnostic criteria (Table S1).^23^ In contrast to most PTHS-related missense variants, which are situated in the bHLH domain (Figure 2), p.Arg389Cys affects the AD2 activation domain. Studies have shown that p.Arg389Cys impairs protein-protein interactions differently than bHLH variants, most likely explaining the atypical presentation.^23^^,^^24^

Surprisingly, DNAm profiling showed that one of the three individuals with p.Arg389Cys was positive for the PHTS episignature. The other two participants clustered together with controls and had suggestively low MVP scores (MVP < 0.1), indicating a PTHS episignature below the cutoff (Figure 3). This inconsistency could be attributed to a potential nested or supplementary PTHS episignature linked to the AD2 activation domain. It is possible that in atypical PTHS, distinct yet partially overlapping pathways might be affected, as opposed to bHLH domain alterations. These findings suggest that, at this point, the PTHS episignature is mainly useable for the correct classification of variants with a loss-of-function effect in *TCF4*, including missense variants affecting the bHLH domain. Further work is needed to confirm and validate these findings.

The specificity of the *TCF4* episignature was further investigated by testing DNAm profiles of two individuals (participant 82 and 83) carrying bi-allelic loss-of-function variants in *CNTNAP2* (OMIM: 604569). These variants underlie an autosomal recessive phenocopy of PTHS, known as Pitt-Hopkins-like syndrome-1 (PTHSL1).^25^ *CNTNAP2* encodes contactin-associated protein 2 (CASPR2), a transmembrane protein categorized within the neurexin family. TCF4 regulates CASPR2 expression, and its functions are mainly related to neuronal development.^24^^,^^25^^,^^26^ Participant 82, carrying the c.1977_1989del (p.Val660Phefs∗9) *CNTNAP2* variant, was positive for the PTHS episignature (Figure 3). However, participant 83, carrying the c.2153G>A (p.Trp718∗) *CNTNAP2* variant, clustered with healthy controls (negative for the PTHS episignature). This individual also carried a large deletion in the 7q35 region (arr[GRCh37]a7q35(147520829–147810263)x1); therefore, it is possible that this CNV is contributing more to DNAm than the *CNTNAP2* variant.^27^^,^^28^ The finding of a positive PTHS episignature in participant 82 could indicate that the CASPR2-related pathway is involved in the PTHS episignature. To confirm an overlapping *CNTNAP2* episignature and improve the specificity of each biomarker, additional cases with PTHSL1 will need to be investigated.

To investigate the relation between our PTHS cohort and 56 previously reported episignature disorders,^29^ we first annotated the genomic location of the PTHS episignature probes in relation to genes and CpG islands (CGIs). We found that the PTHS DMPs predominantly map within coding regions of genes and CGI shore regions (within 0–2 kb of a CGI boundary) as well as regions outside CGIs. Comparing PTHS to the other 56 episignature disorders, we observed an overlap in the mapping to intergenic regions. Most of the PTHS episignature probes are located in promoter and promoter + regions, compared to background probes. In relation to CGIs, PTHS episignature probes were more located in inter-CGIs and CGIs and less in shore and shelf regions (see Figure S5).

**Figure 4:**
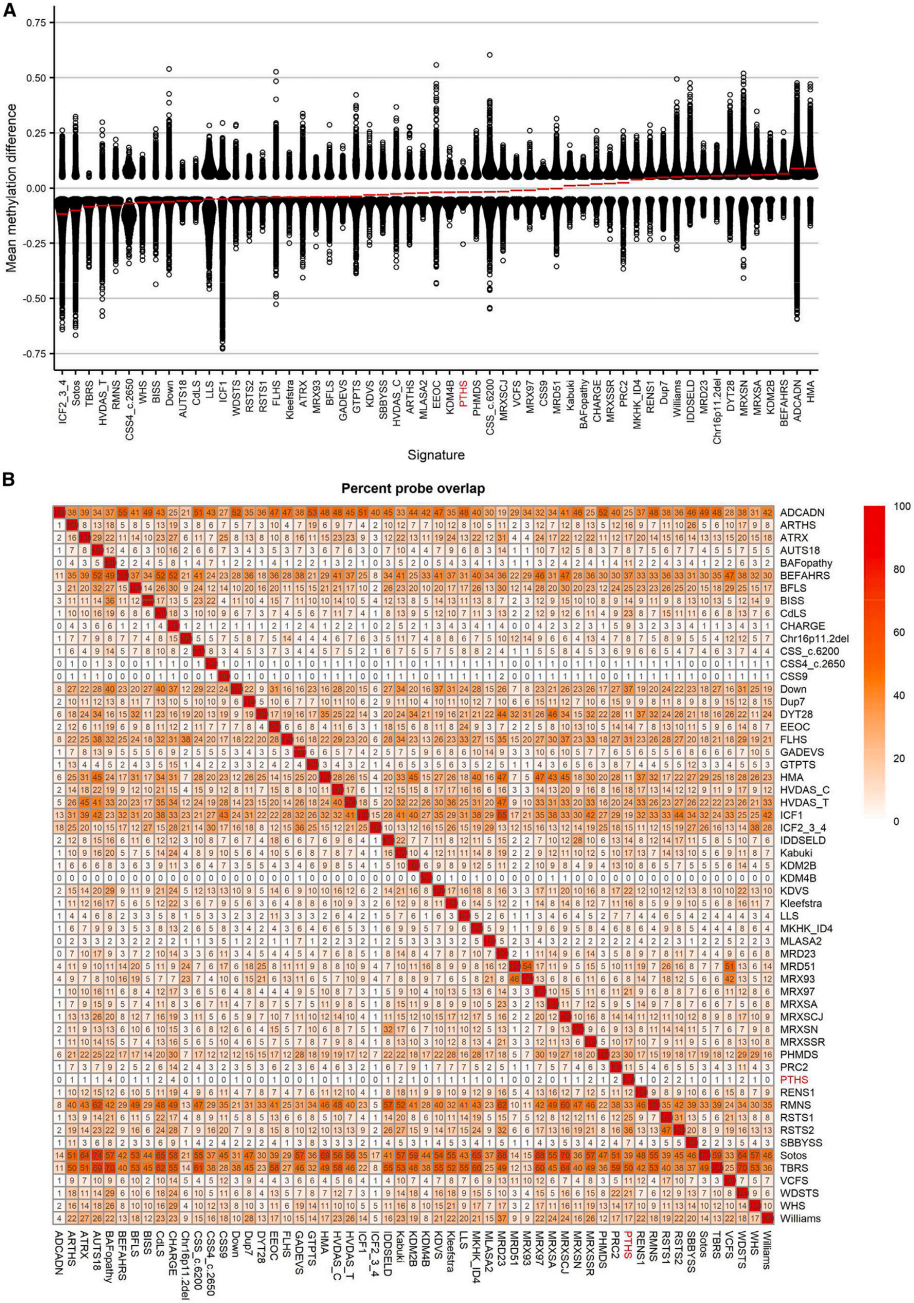
Relationships between the PTHS cohort and 56 other EpiSign disorders (A) Methylation Profiles - Methylation profiles of all differentially methylated positions (DMPs) with a false discovery rate (FDR) <0.05 are presented for each cohort. The probes are sorted by their mean methylation values, with each circle representing an individual probe and red lines indicating the mean methylation levels. (B) Shared probes heatmap. A heatmap displays the percentage of probes shared between each paired cohort. The colors within the heatmap indicate the proportion of probes from the y axis cohort also present in the x axis cohort’s probes, offering insights into the overlap of methylation patterns between different cohorts.

We then investigated the overlap of the genome-wide DNAm changes in PTHS cases with pathogenic *TCF4* variants and CNVs involving *TCF4*, alongside 56 previously reported episignatures.^29^ Clustering analyses was performed using the top 500 DMPs for each cohort. For cohorts with fewer than 500 DMPs, the total number of DMPs was used to assess the similarity in genome-wide DNAm profiles. Our analysis revealed a predominantly hypermethylated profile in PTHS (Figure 4A). Notably, PTHS exhibited the highest percent of DMPs overlapping with BRG1/BRM-associated factor (BAFopathy) (4%, including *ARID1A*, *ARID1B*, *SMARCB1*, *SMARCA2*, and *SMARCA4*) and coloboma, heart anomaly, choanal atresia, retardation, genital and ear anomalies (CHARGE) (4%, *CHD7*) (Figures 4B and S6). This observed overlap can be attributed to the extensive DMPs present in the episignatures of BAFopathy and CHARGE. Interestingly, many other episignatures also overlap with those of BAFopathy and CHARGE episignatures.^29^

**Figure 5:**
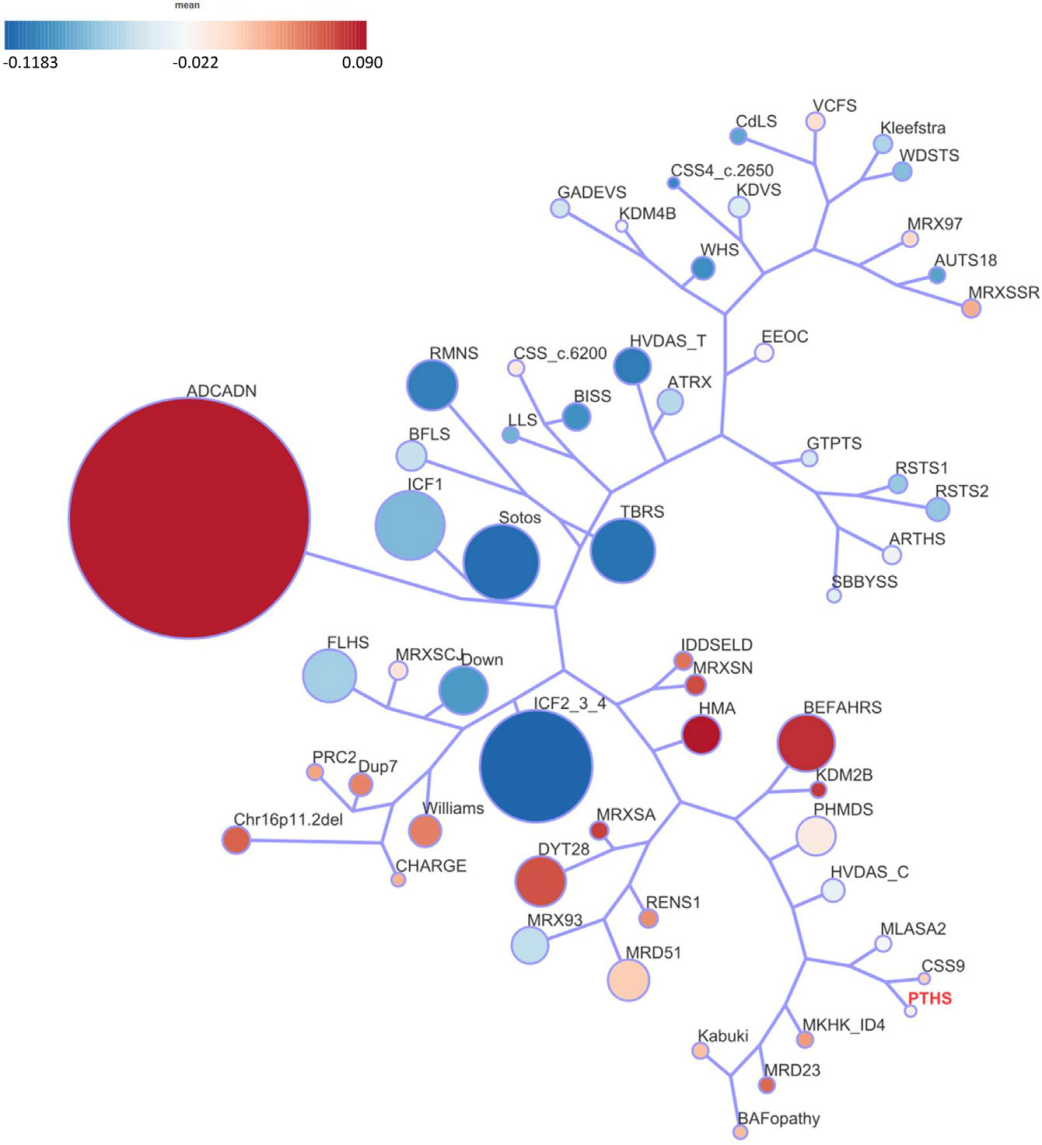
Tree and leaf visualization of episignatures by mean methylation status of each DMP per syndrome This figure illustrates a tree and leaf visualization of episignatures, portraying the interrelationships among all 57 cohorts. To generate this visualization, Euclidean clustering was employed using the top 500 DMPs for each cohort. Cohort samples were aggregated based on the median methylation values of each DMP within the group. In this representation, each leaf node represents to a specific cohort, with node sizes indicating the relative number of selected DMPs for that cohort. The colors of the nodes reflect the mean methylation difference. This visualization offers valuable insights into the clustering and similarities of methylation patterns across different cohorts, providing valuable information about the epigenetic profiles and their relationships.

Subsequently, we visually represented the interrelations among all 57 cohorts by examining DMP overlap and direction of effect, utilizing a binary tree (Figure 5). Each node in this representation corresponds to a specific cohort. Upon analysis, we observed that PTHS formed a hypermethylation closely linked to Coffin-Siris syndrome, which is associated with *SOX11* (OMIM: 600898) pathogenic variants (Figure 5). Interestingly, the *SOX11*-associated Coffin-Siris episignature exhibited a slightly more hypermethylated profile compared to the PTHS episignature. The presence of shared DMPs in these two cohorts suggests an underlying biological similarity. Notably, *TCF4* and *SOX11* are known to have a documented biochemical interaction, demonstrated through overexpression in HEK293 cells They are believed to work in conjunction to regulate commissure formation while also playing a role in the transcriptional control of genes implicated in this process.^6^

In summary, this study has successfully identified a DNAm episignature for PTHS, facilitating molecular testing and the reclassification of *TCF4* VUSs in individuals exhibiting signs of PTHS without the requirement additional tissue analyses. The PTHS episignature demonstrates high sensitivity to *TCF4* pathogenic variants in *TCF4* with a loss-of-function effect including missense variants located within the bHLH domain underlying the most common pathophysiological mechanisms of PTHS. Exploratory episignature mapping of atypical PTHS and PTHSL1 samples, despite the relatively limited sample, offered valuable insights into possibly common affected pathways and identified the possibility of additional or nested episignatures. Greater efforts are needed to ensure full episignature coverage of atypical PTHS and DNAm changes related to PTHS phenocopies.

## Data and code availability

The data supporting the current study have not been deposited in a public repository to protect individual confidentiality but are available from the corresponding author upon request.

## Supplemental information

Data S1. Tables S1–S4

Document S2. Article plus supplemental information
