# Supplementary material for: DNA methylation episignature and comparative epigenomic profiling for Pitt-Hopkins syndrome caused by TCF4 variants
Source: HGG Adv. 2024 Apr 2;5(3):100289. doi: 10.1016/j.xhgg.2024.100289 (PMC11087720; doi:10.1016/j.xhgg.2024.100289)
Supplement: Document S1. Figures S1–S6 and supplemental methods [file mmc1.pdf]

HGGA, Volume *5*

Supplemental information

DNA methylation episignature

and comparative epigenomic profiling

for Pitt-Hopkins syndrome caused by *TCF4* variants

Liselot van der Laan, Peter Lauffer, Kathleen Rooney, Ananília Silva, Sadegheh Haghshenas, Raissa Relator, Michael A. Levy, Slavica Trajkova, Sylvia A. Huisman, Emilia K. Bijlsma, Tjitske Kleefstra, Bregje W. van Bon, Özlem Baysal, Christiane Zweier, María Palomares-Bralo, Jan Fischer, Katalin Szakszon, Laurence Faivre, Amélie Piton, Simone Mesman, Ron Hochstenbach, Mariet W. Elting, Johanna M. van Hagen, Astrid S. Plomp, Marcel M.A.M. Mannens, Mariëlle Alders, Mieke M. van Haelst, Giovanni B. Ferrero, Alfredo Brusco, Peter Henneman, David A. Sweetser, Bekim Sadikovic, Antonio Vitobello, and Leonie A. Menke

Supplemental

Figures and Methods

Supplemental figures

A

B

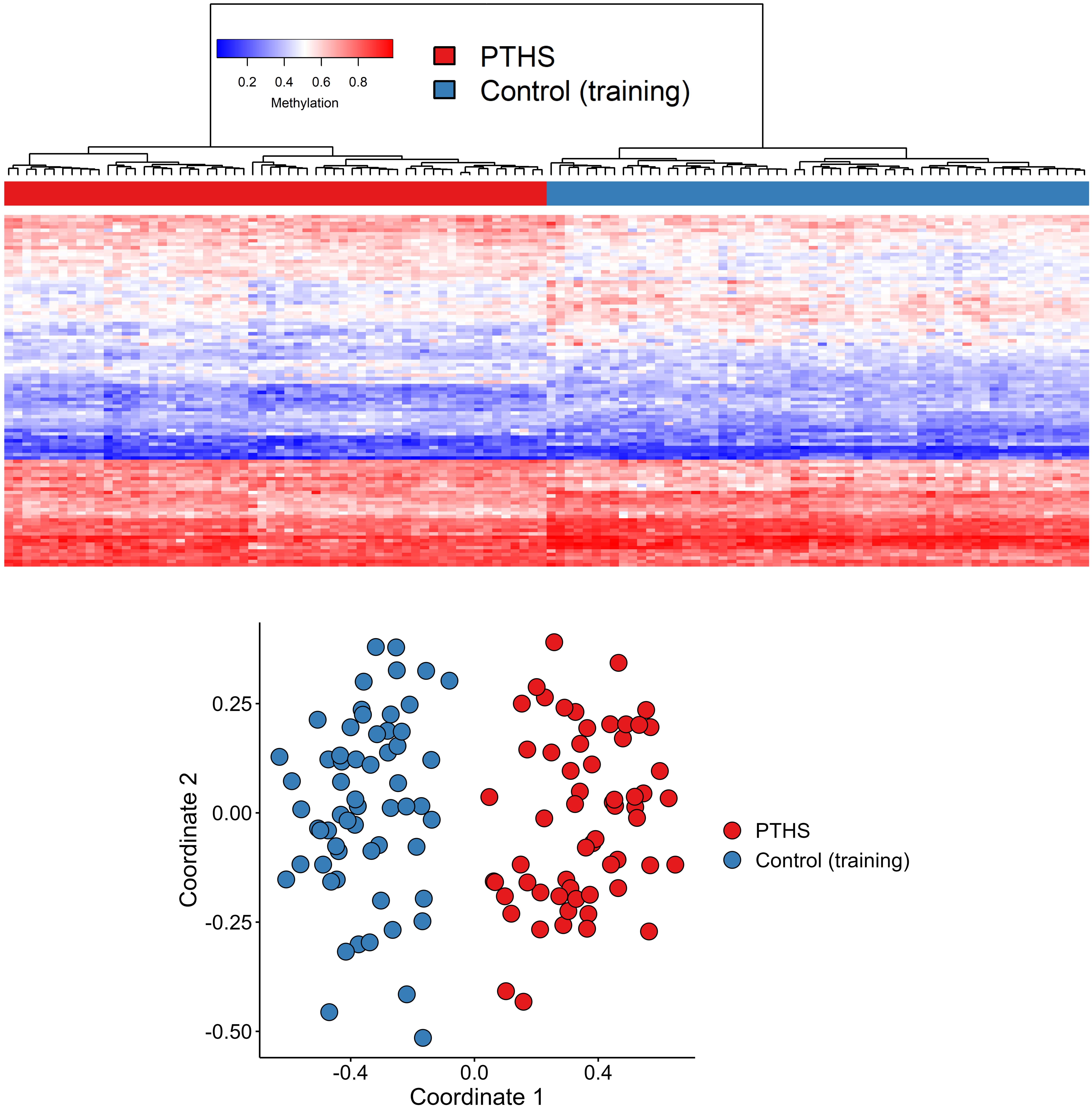

Figure S1; Pitt Hopkins Syndrome Episignature Discovery Cohort. (A) Euclidean hierarchical clustering heatmap, where each column represents one TCF4 discovery case or control, and each row corresponds to a probe selected for the episignature. The heatmap visually separates the cases (in red) from controls (in blue). (B) Multidimensional scaling (MDS) plot illustrating the distinct clustering of TCF4 cases and controls.

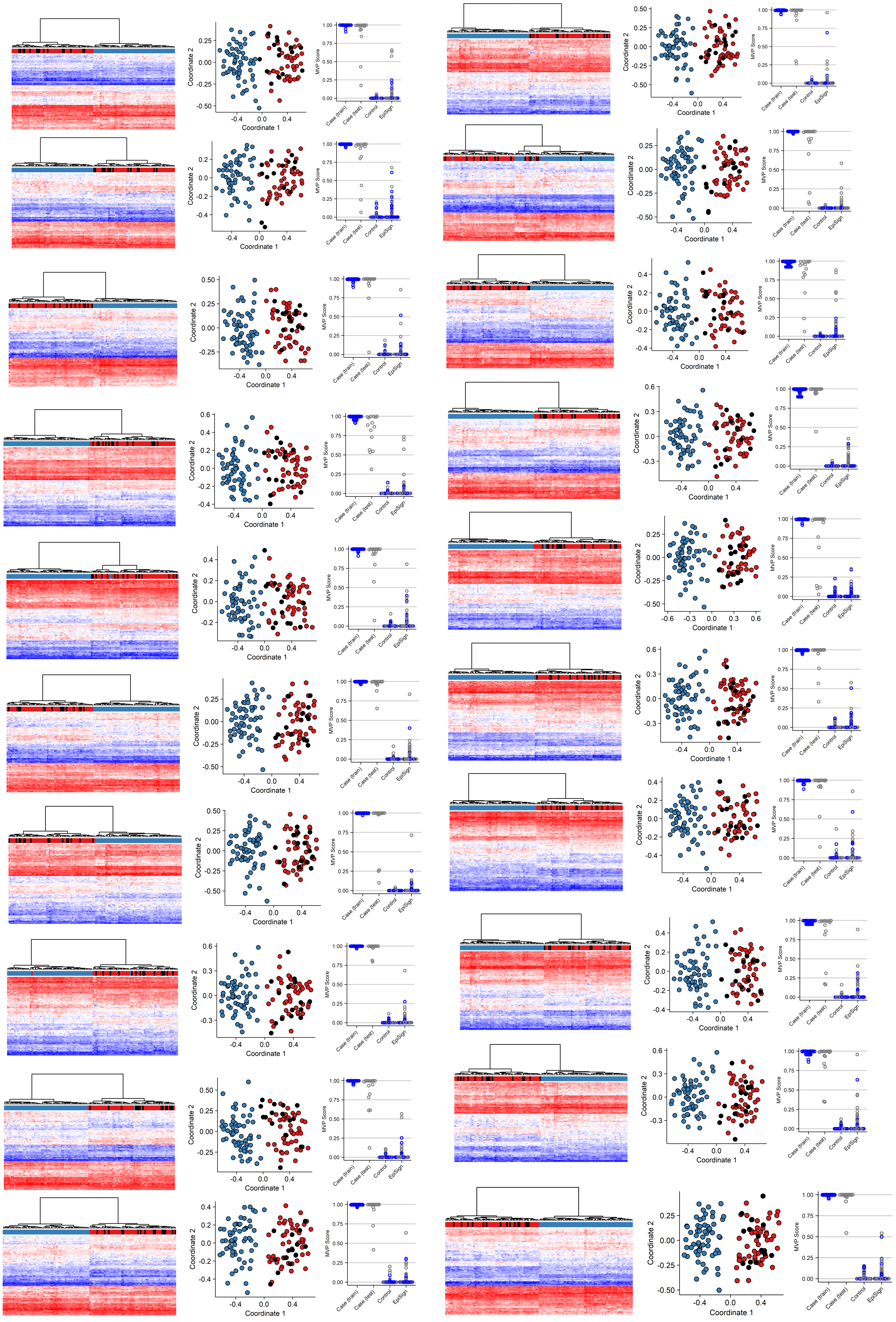

Figure S2; Discovery Cohort Leave-25%-Out Cross Validation. Twenty rounds of leave-25%-out cross-validation are presented. In each set, 15 test case samples (in black) are used for testing, while the remaining TCF4 cases used for episignature training are shown in red, and control training samples are in blue in both the heatmap and MDS plots. The last plots showcase the Methylation Variant Pathogenicity (MVP) scores of the Support Vector Machine (SVM) classifier model trained using the selected TCF4 episignature probes from training cases, 75% of controls, and other EpiSign samples (in blue). The remaining 25% of controls and other disorder samples were used for testing, alongside the TCF4 cases (in grey).

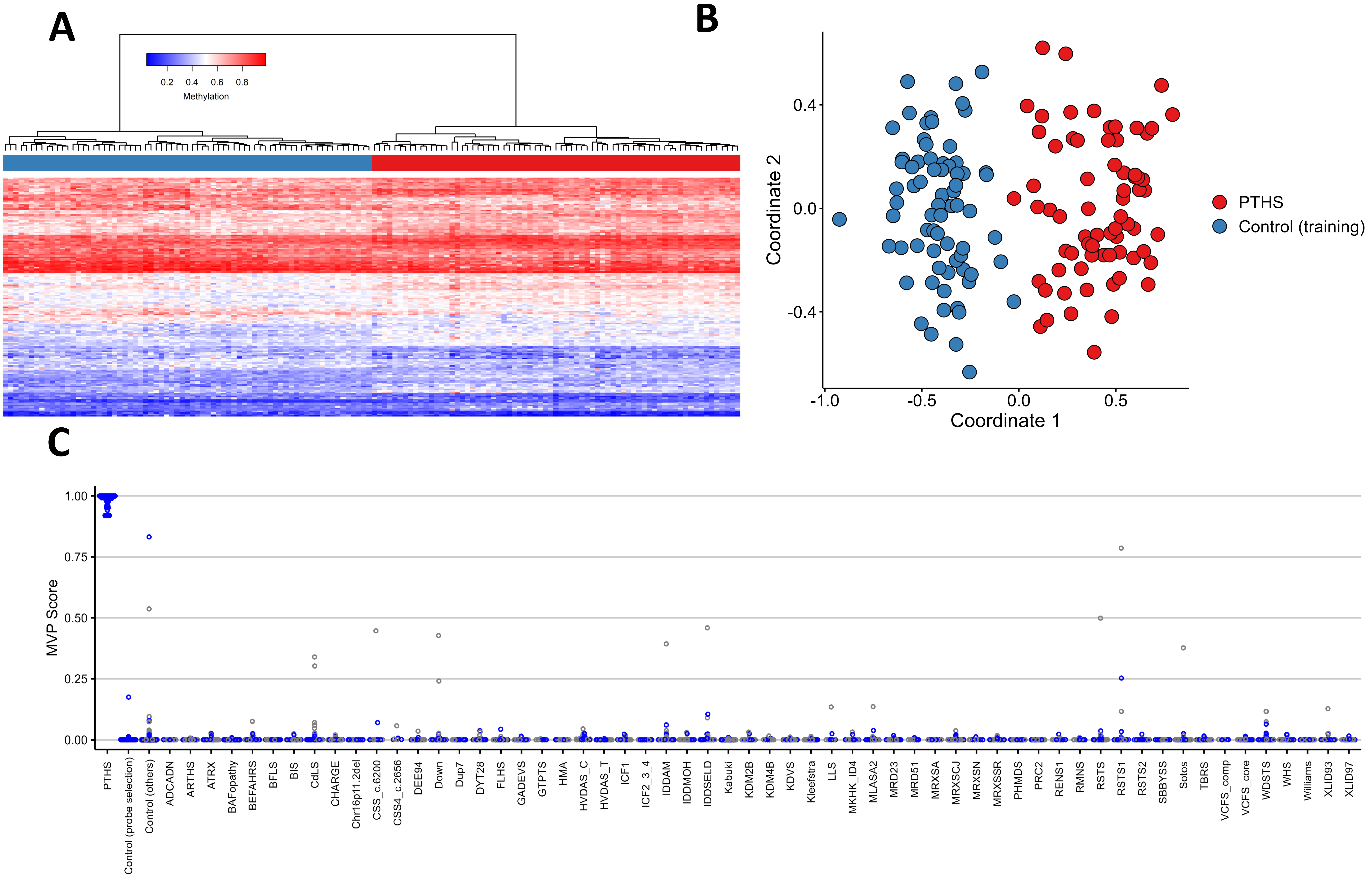

Figure S3; Pitt Hopkins Syndrome Episignature Discovery and Validation Cohort Combined Training. (A) Euclidean hierarchical clustering heatmap representing TCF4 discovery cases and controls, with each column corresponding to one case or control, and each row representing a probe selected for the episignature. The heatmap demonstrates a clear separation between cases (in red) and controls (in blue). (B) MDS plot shows the segregation of TCF4 cases and controls. (C) SVM classifier model trained using selected PTHS episignature probes, 75% of controls, and 75% of other neurodevelopmental disorder samples (in blue). The remaining 25% of controls and 25% of other disorder samples were used for testing (in grey). The plot illustrates that PTHS samples exhibited MVP scores >0.75.

.

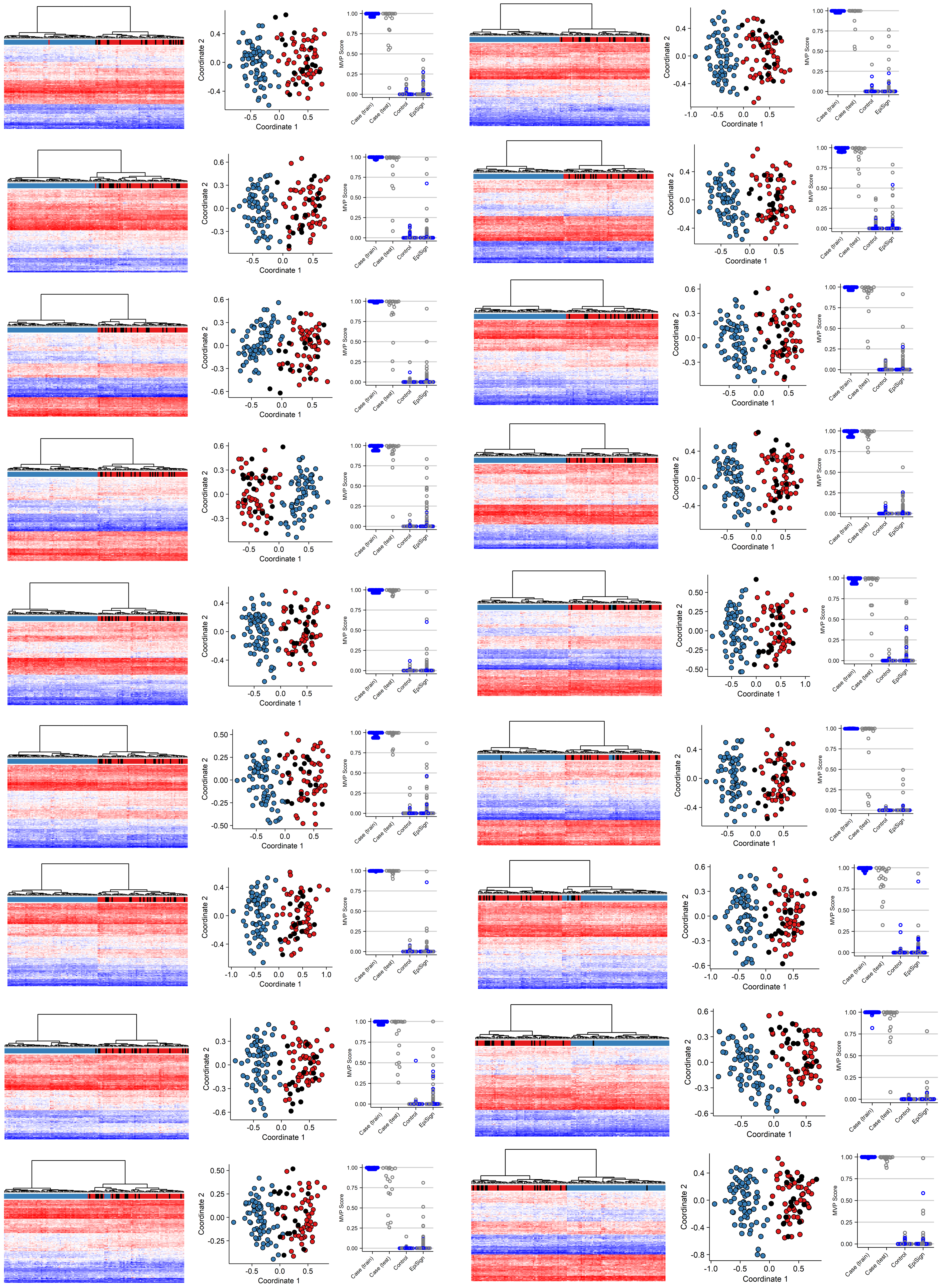

Figure S4; Combined Discovery and Validation Cohort Leave-25%-Out Cross Validation. Similar to Supplemental Figure 2, this figure presents twenty rounds of leave-25%-out cross-validation. In each set, 18 test case samples (in black) are used for testing, and the remaining TCF4 cases used for episignature training are shown in red, along with control training samples in blue in both the heatmap and MDS plots. The last plots demonstrate the MVP scores of the SVM classifier model trained using the selected TCF4 episignature probes from training cases, 75% of controls, and other EpiSign samples (in blue). The remaining 25% of controls and other disorder samples are used for testing, alongside the TCF4 cases (in grey).

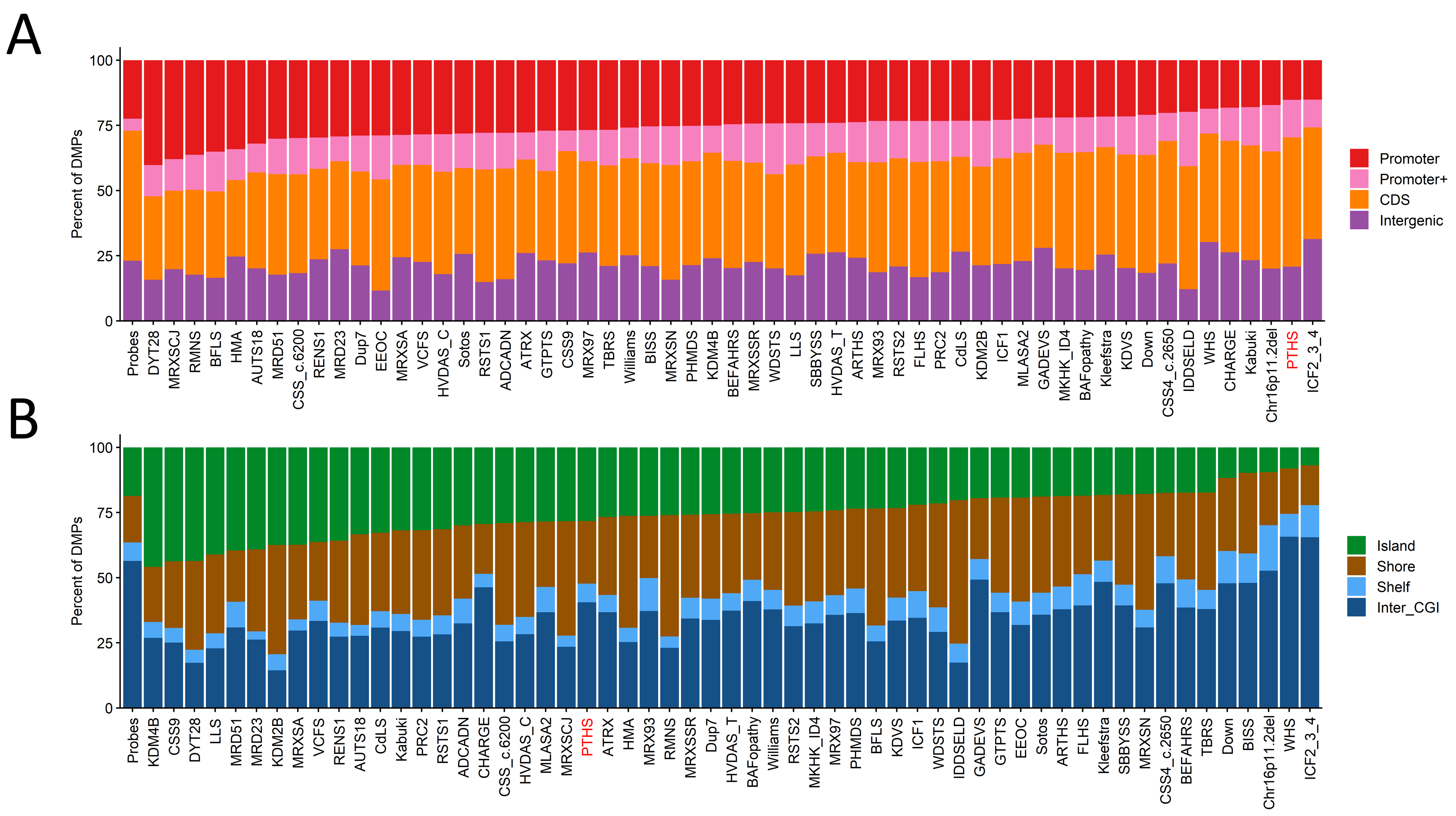

Figure S5. Differentially Methylated Probes (DMPs) Annotated . (A) DMPs in relation to genes. (B) DMPs in CpG islands. Promoter (0-1 kb upstream of the transcription start site), Promoter+ (1-5 kb upstream of the TSS), CDS (coding sequence), Intergenic (other genome regions). Island (CpG islands), Shore (within 0-2 kb of a CpG island boundary), Shelf (within 2-4 kb of a CpG island boundary), Inter_CGI (other genome regions).

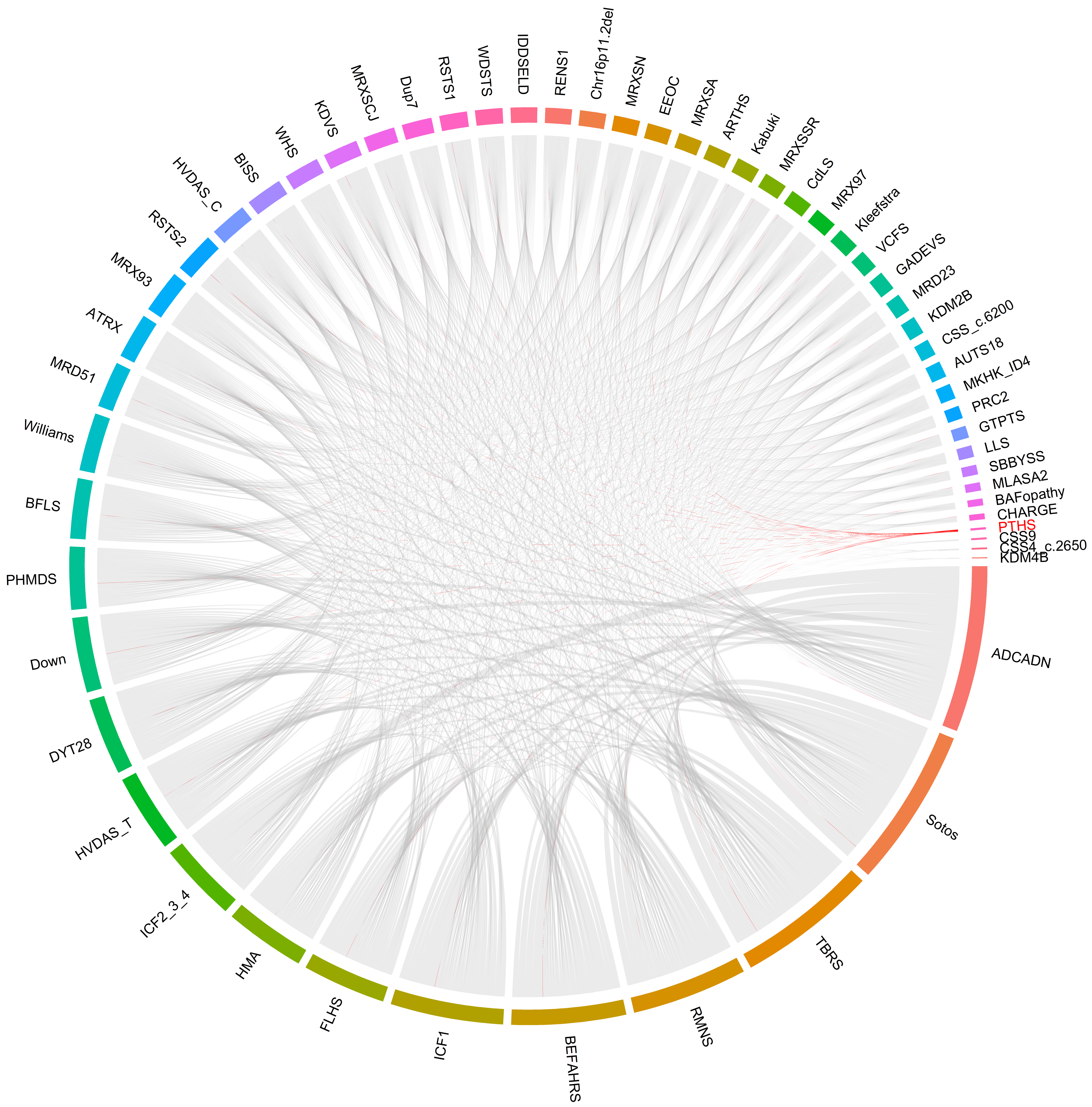

Figure S6. Differentially Methylated Probes (DMPs) Shared Between PTHS Cohort and 56 Other Episignatures on EpiSign™. This Circos plot visually depicts the probes shared between each pair of cohorts, with the thickness of connecting lines indicating the number of shared probes. Connections involving the PTHS cohort are highlighted in red.

Supplemental methods

DNA methylation data

Bisulfite converted genomic DNA, isolated from peripheral blood was applied to the Infinium methylation EPIC Bead Chip (San Diego, CA) array according to manufacturer's protocol. We used the minifi package (version 1.44.0) to generate the intensity data files (IDATS) and import them into R (version 4.2.3) [1]. Standard preprocessing for Illumina microarrays were implemented. First, we preformed background correction and normalization. Then the quality control was preformed, which included the evaluation of density plots and checking for discordance in recorded and predicted sex and age. Finally, probes were filtered based on the removal of the following; probes that overlap with single-nucleotide variation, probes that are cross-reactive, probes specific to regions on the X or Y chromosomes and probes with detection p-value >0.1. the final number of probes after removal was 772557.

DNA methylation analyses

DNA methylation analyses were performed according to our previously published work [2, 3]. To summarize, first the matched controls from the EKD (EpiSign knowledge database) bases on sex, age, batch and array type were selected with the package [4] however, samples in the EKD with batch effect and/or >5% probe failure were excluded. With principal component analyses (PCA) we examined the training cohort and the matched case-control samples for data structure and outliers. Then feature selection was preformed using matched cases and controls. With the limma package (version 3.54.2) [5] differential methylation analysis was performed with linear regression fitting. Methylation beta values were used as predictors and labels as response, with the model adjusted for estimated blood cell counts as confounding variables. The empirical bayes method was applied to control for false discoveries and adjusted using the Benjamini-Hochberg procedure to compute the moderated t-statistics and P-values. Next, the separate clustering of cases and controls was investigated with heatmaps and multidimensional scaling (MDS) with the package ggplot2 (version 3.1.3). The best assessed clustering by parameter values was selected. Finally, leave-25%-out cross validation and unsupervised clustering results were performed to investigate the reproducibility of the episignature.

To identify differentially methylated regions (DMRs) we also performed analyses with the R package DMRcate (version 2.12.0) [6]. The settings were defined as at least CpGs within 1 kb distance of each other and at least 0.05 absolute mean methylation difference between cases and controls. We further filtered the results with the Fisher P-value cutoff of 0.01.

Prediction model

To investigate the sensitivity and specificity of the PTHS episignature cohort we used the classifier and all the episignature probes. Our support vector machine (SVM) model was trained with the package e1071 (version 1.7-13) using the selected features and the matched controls and cases as training data. Next, cases (known to have an episignature, unaffected samples and training controls) in the EKD were included, the other 25% were used as testing. We repeated these four times, so that every sample was used as a testing sample one time. The average SVM was then used, also named the methylation variant pathogenicity (MVP) score.

Overlap of the PTHS Genome-Wide DNA Methylation Profile with Other Neurodevelopmental Disorders on EpiSign™

The functional annotation and EpiSignTM cohort comparison were performed based on previously published articles [7-9]. To summarize, we assessed the percentage of DMPs shared between the PTHS episignature and the other 56 neurodevelopmental disorder episignatures on the EpiSignTM clinical classifier were assessed and heatmaps and circos plots produced. With the package pheatmap (version 1.0.12), heatmaps were plotted and the circos plots were generated with the R package circlize (version 0.4.15)[10]. We preformed clustering analysis to investigate relationships between all the cohorts with known episignatures. To generate a tree and leaf plot to show the distance and similarities between the cohorts we used the package TreeAndLeaf (version 1.6.1) [11]. To discover the genomic locations of the selected DMPS in our cohort, probes were annotated in relation to the CpG islands (CGIs) and genes with the R package annotatr (version 1.20.0) [12] with AnnotationHub (version 3.2.2) as described previously by Levy et al. [7].

Supplemental references

1. Aryee, M.J., et al., Minfi: a flexible and comprehensive Bioconductor package for the analysis of Infinium DNA methylation microarrays. Bioinformatics, 2014. 30(10): p. 1363-9.
2. Levy, M.A., et al., Novel diagnostic DNA methylation episignatures expand and refine the epigenetic landscapes of Mendelian disorders. HGG Adv, 2022. 3(1): p. 100075.
3. Aref-Eshghi, E., et al., Diagnostic Utility of Genome-wide DNA Methylation Testing in Genetically Unsolved Individuals with Suspected Hereditary Conditions. Am J Hum Genet, 2019. 104(4): p. 685-700.
4. Ho, D., et al., MatchIt: Nonparametric Preprocessing for Parametric Causal Inference. Journal of Statistical Software, 2011. 42(8): p. 1 - 28.
5. Ritchie, M.E., et al., limma powers differential expression analyses for RNA-sequencing and microarray studies. Nucleic Acids Res, 2015. 43(7): p. e47.
6. Peters, T.J., et al., De novo identification of differentially methylated regions in the human genome. Epigenetics Chromatin, 2015. 8: p. 6.
7. Levy, M.A., et al., Functional correlation of genome-wide DNA methylation profiles in genetic neurodevelopmental disorders. Hum Mutat, 2022.
8. van der Laan, L., et al., Episignature Mapping of TRIP12 Provides Functional Insight into Clark–Baraitser Syndrome. International Journal of Molecular Sciences, 2022. 23(22): p. 13664.
9. Rooney, K., et al., DNA methylation episignature and comparative epigenomic profiling of HNRNPU-related neurodevelopmental disorder. Genet Med, 2023. 25(8): p. 100871.
10. Gu, Z., et al., circlize Implements and enhances circular visualization in R. Bioinformatics, 2014. 30(19): p. 2811-2.
11. Cardoso, M.A., et al., TreeAndLeaf: an R/Bioconductor package for graphs and trees with focus on the leaves. Bioinformatics, 2022. 38(5): p. 1463-1464.
12. Cavalcante, R.G. and M.A. Sartor, annotatr: genomic regions in context. Bioinformatics, 2017. 33(15): p. 2381-2383.
